# Effect of the *TERT* mutation on the prognosis of patients with urothelial carcinoma: a systematic review and meta-analysis

**DOI:** 10.1186/s12894-023-01349-9

**Published:** 2023-11-02

**Authors:** Hui Shuai, Xi Duan, Jun-Jie Zhou, Yuan Liu, Tao Wu

**Affiliations:** 1 Department of Urology, Affiliated Hospital of North Sichuan Medical College, Wenhua Road 57, Shunqing District, Nanchong, Sichuan 637000 People’s Republic of China; 2 Department of Dermatology, Affiliated Hospital of North Sichuan Medical College, No. 1 Maoyuan South Road, Shunqing, Nanchong, Sichuan 637000 People’s Republic of China

**Keywords:** Urothelial carcinoma, *TERT*, Prognosis, Survival, Meta-analysis

## Abstract

**Background:**

Telomerase reverse transcriptase (*TERT*) mutation represents the most prevalent genetic mutation found in urothelial carcinoma (UC) and holds potential as a prognostic indicator for tumor outcomes. However, the association between *TERT* mutation and prognosis in UC patients remains poorly elucidated due to conflicting findings in existing literature. Therefore, this study aimed to investigate the effect of the *TERT* mutation on the survival of UC patients.

**Methods:**

We systematically searched the PubMed, Embase, and Cochrane Library databases for studies that investigated the relationship between the *TERT* mutation and the prognosis of UC patients. Endpoints included the 2-year and 5-year recurrence-free survival (RFS) and overall survival (OS). The Newcastle–Ottawa Scale (NOS) tool was used to assess the risk of bias in the included studies. Review Manager 5.3 was used for the meta-analysis.

**Results:**

Nine studies with a total of 1,552 patients were included in the analysis. Two studies were prospective, and seven were retrospective. The *TERT* promoter mutation was associated with a lower 2-year OS (relative risk [RR] = 0.92, 95% confidence interval [CI] 0.86–0.98; *P* = 0.007) and a lower 5-year OS (RR = 0.80, 95% CI 0.68–0.94; *P* = 0.008) compared with the *TERT* wild type. However, no significantly differences were found between two groups in terms of HR for OS (hazard ratio [HR] = 1.29, 95% CI 0.80–2.08; *P* = 0.29). Furthermore, we investigated the differences in RFS and disease-specific survival (DSS) between the two groups.

**Conclusion:**

The *TERT* mutation increases the risk of death and decreases the survival time of UC patients. *TERT* may be a valuable marker with individual prognostic value.

## Introduction

Urothelial carcinoma (UC) is the most common malignant tumor of the urinary system, with a high incidence of morbidity and mortality [1, 2]. It can be found anywhere in the urinary tract, from the renal pelvis to the urethra [3]. Known tumor markers such as p53, Aurora-A, and plasma fibrinogen are associated with the diagnosis and prognosis of UC [4, 5]. But they are also common in other tumors and lack specificity. Moreover, finding a biomarker to accurately diagnose and predict the prognosis of UC is challenging due to its molecular heterogeneity [6]. Therefore, it is imperative to investigate the cancer biology and pathogenesis in order to ascertain the prognosis of UC.

Telomeres are specialized structures located at the ends of chromosomes in eukaryotic cells. They are fundamentally DNA–protein complexes with significant biological functions, such as stabilizing chromosomes, preventing DNA fusion and degradation, protecting chromosomal structural genes, and regulating normal cell growth. Telomerase is a ribonucleoprotein that synthesizes telomere DNA at the ends of chromosomes, thereby compensating for terminal replication and allowing cells to proliferate indefinitely [7–9]. Telomerase is activated in the majority of human cancers, including bladder cancer and some urogenital tumors. Increased telomerase activity is attributed to the transcriptional regulation of *TERT* and is regarded as a hallmark of malignancy in humans [10, 11]. The *TERT* mutation is also the most common gene mutation in UC [3]. However, the association between *TERT* mutation and prognosis in UC patients remains poorly elucidated. Most studies have found that the *TERT* mutation shortens patient survival, which is related to disease progression and recurrence [1, 3, 12–15]. On the contrary, Jenny et al. reported that UC patients harboring the *TERT* mutation exhibit elevated rates of recurrence-free survival (RFS) at the 2-year and 5-year marks [6]. Likewise, *TERT* mutation improved PFS (HR 0.38, *P* = 0.012) and OS (HR 0.32, *P* = 0.037) in one study [16]. No difference was found between two groups regarding PFS and OS in Neal’s study [17].

Therefore, we conducted a systematic review and meta-analysis to determine the impact of the TERT mutation on the survival of UC patients and to aid in clinical treatment planning.

## Methods

The systematic review and meta-analysis were based on the Preferred Reporting Items for Systematic Reviews and Meta-Analyses (PRISMA) statement [18], and the study has been registered on the International Prospective Register of Systematic Reviews (PROSPERO: CRD42023430667).

### Eligibility criteria

Eligibility criteria were formulated using the specific population, intervention, comparison, outcomes, and study design (PICOS) framework. This review included studies that fulfilled the following criteria: (P): adults aged over eighteen with UC; (I): gene sequencing identified the *TERT* mutant type; (C): gene sequencing identified the *TERT* wild type; (O): OS, RFS, and DSS; (S): retrospective and prospective cohort studies.

Case series, surveys, letters, editorial comments, reviews, and animal studies were not included. In addition, studies without original data, those that did not explicitly report HR or Kaplan–Meier curves, and articles in languages other than English were excluded.

### Information sources, search strategy, and selection process

A systematic search was conducted using Embase, PubMed, and the Cochrane Library. The search terms used were: (Telomerase OR *TERT* OR Telomerase Reverse Transcriptase OR Reverse Transcriptase; Telomerase OR Transcriptase; Telomerase Reverse OR Telomerase Catalytic Subunit) AND (Carcinoma; Transitional Cell OR Transitional Cell Carcinoma OR Transitional Cell Carcinomas OR Urothelial Carcinoma OR Urothelial Carcinomas). The search results were limited to humans. Studies published between January 1, 1990, and February 1, 2023, were included. Studies that meet our PICOS criteria were included.

### Data collection process and data items

Two authors extracted data from the nine included studies. Data extracted included study type (prospective or retrospective study), first author, study duration, pathological type of tumor, number of patients, sex ratio, follow-up time, survival outcome, 2-year or 5-year survival rate (OS, RFS, and DSS), HR with 95% CI, and data source. The Engauge Digitizer was used to extract the survival rate from the Kaplan–Meier curves. We calculated HR for studies that did not present a 95% confidence interval (CI) employing the methodologies outlined in the literature of Jayne F. Tierney [19]. Three outcome measures were analyzed based on data availability and clinical correlation: OS, RFS, and DSS. Where disease-free survival (DFS) and failure-free survival were presented, these outcomes were deemed equivalent to RFS. The results could not be extracted if the study authors chose to stratify results based on a specific subgroup of the study rather than report results for the entire population.

### Risk of bias assessment

The Newcastle–Ottawa Scale (NOS) [20, 21] was used to assess the quality of all studies. The NOS checklist includes three quality parameters: population selection (four points), comparability of cohorts (two points), and assessment of outcome for cohort studies (three points). Each study received a score ranging from zero to nine. Studies with a score of seven or higher were considered high-quality articles.

### Synthesis methods

The meta-analysis included retrospective and prospective cohort studies and was performed using Review Manager 5.3 (Cochrane Collaboration, Oxford, UK). We pooled clinical effect estimates using the hazard ratio (HR), relative risk (RR), and their respective 95% CIs. The statistical significance level was set at *P* < 0.05. The Mantel–Haenszel effects model and inverse-variance effects model were used to combine the trials. We calculated and depicted forest plots with a 95% CI. The *I*^2^ test and Cochran’s Q test were used to assess the heterogeneity. Statistical heterogeneity was indicated by *P* < 0.1 in the Cochran’s Q test and *I*^2^ > 50% in the I^2^ test. If heterogeneity existed, a random effect model was adopted; otherwise, a fixed effect model was adopted. *I*^2^ values of 25%, 50%, and 75% indicate low, moderate, and high levels of inconsistency, respectively [22]. Various Kaplan–Meier curves described in the original literature were used to calculate the survival rate and HR with a 95% CI. Further sensitivity analyses were conducted to reduce heterogeneity and confirm the reliability of our findings.

## Results

### Study selection, characteristics, and risk of bias

**Fig. 1:**
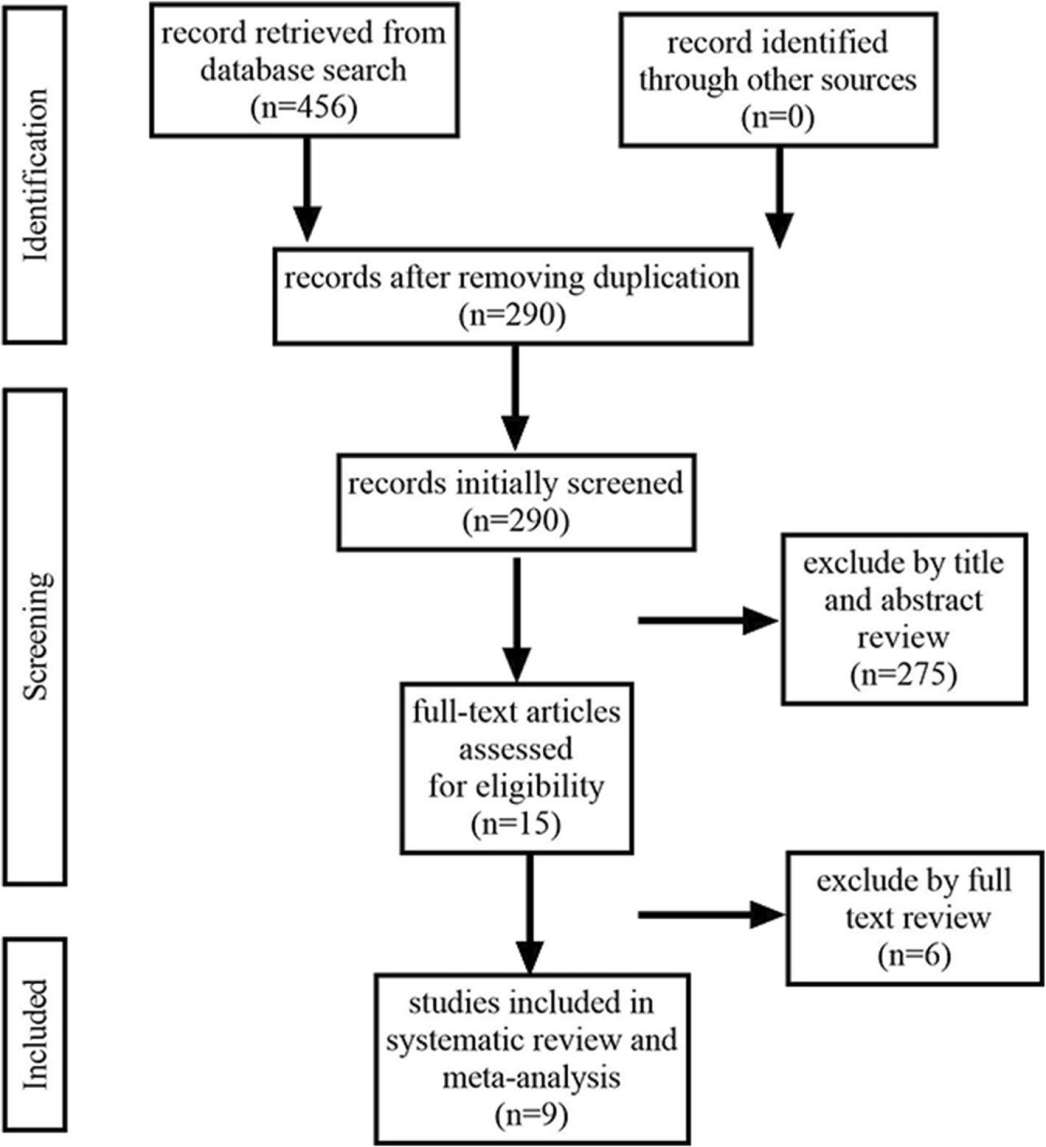
Flowchart illustrating the major steps of the review process in accordance with the Preferred Reporting Items for Systematic Reviews and Meta-analyses (PRISMA) statement

We found 455 articles, of which nine [1, 3, 6, 12–17] were selected for further analysis. Figure 1 depicts the search process (PRISMA flowchart). Two studies were prospective, and seven were retrospective. There were 1,552 participants. The follow-up period ranged from 5 to 25 years. Four studies focused on bladder cancer, one on upper tract urothelial cancer (UTUC), and four on urothelial carcinoma. Table 1 provides an overview of the patients and details of our study population. Furthermore, in the cohort study by Nakanishi et al. [14], DFS was the time between the initial radical operation and the subsequent appearance of recurrence. In this study, DFS is considered to be equivalent to RFS.

**Table 1:** Characteristics of the included studies

Author	Year	Country	design	Accrual	Histology	FU	Mutation detection	Survival outcomes	Participants (M/W)	Tumor grade (Low/High)	Tumor stage (Ta-1/T2-4)	Age (Median; range)	Gender (male%)
Rachakonda	2013	Germany	prospective	1995–2010	BC	15y	PCR Sanger	OS	186/93	118/209	219/108	72.8(65.4–79.4)^a^	NR
Nakanishi	1999	Japan	retrospective	1970–1995	UTUC	300 m	In situ hybridization	DFS	42/80	77/51	NR	66(34–84)	71.9
								OS	44/84				
Yang CH	2008	China	retrospective	1995–2010	UC	10y	streptavidin–biotin method	RFS	50/44	13/81	NR	67(21–83)	41.5
Sumit	2017	America	retrospective	NR	UC	200 m	MSK-IMPACT	OS	266/122	35/353	38/84	66(58–74)	NR
								DSS	266/122				
								MFS	198/91				
Song Wu	2013	China	retrospective	NR	BC	120 m	Sanger	OS	120/96	NR	56/64	NR	NR
Ismail	2015	Germany	prospective	1995–2010	BC	10y	PCR Sanger	OS	102/79	87/127	148/66	72.8(65.4–79.4)^a^	NR
Jenny	2019	Germany	retrospective	2012–2018	BC	1600d	PCR	OS	63/12	25/50	50/25	75(49–97)	78.7
								DSS	63/12				
								RFS	63/12				
Ivan	2021	America	retrospective	2014–2020	UC	6y	NR	OS	47/31	NR	NR	71(66–76)	62.8
Neal	2023	America	retrospective	2014–2021	UC	7y	NR	PFS	64/49	NR	NR	67(35–85)	76%
								OS	64/49				

*FU* Follow-up, *M/W* Mutant/wild-type, *NR* Not reported, *MSK-IMPACT* Memorial Sloan Kettering Integrated Mutation Profiling of Actionable Cancer Targets, *PCR* Polymerase Chain Reaction, ^a^these numbers are median (inter-quartile range)

Table 2 presents the variables from the included studies. The assessment of the effect of the *TERT* mutation on the prognosis of UC patients varied but included at least one of the following: OS, RFS, and DSS.

**Table 2:** The variables of the included studies

Stusy	Survival outcomes	2-yr		5-yr		HR	LCI	UCI	Data source	NOS score
		Mutation	Wild	Mutation	Wild					
Rachakonda 2013	OS	0.80	0.81	0.68	0.73	1.34	0.81	2.23	Published	9
Nakanishi 1999	OS	0.66	0.85	0.57	0.79	2.50	1.26	4.96	Estimated^a^	8
	RFS	0.50	0.70	0.39	0.65	1.94	1.14	3.31		
Yang CH 2008	RFS	0.81	0.91	0.71	0.84	NA	NA	NA	Estimated	7
Sumit 2017	OS	0.69	0.79	0.57	0.68	2.31	1.46	3.65	Published	8
	DSS	0.69	0.79	0.58	0.69	2.23	1.41	3.53		
	MFS	0.62	0.68	0.52	0.58	1.63	1.05	2.53		
Song Wu 2013	OS	0.84	0.98	0.66	0.98	7.61	1.69	34.19	Estimated	8
Ismail 2015	OS	0.68	0.77	0.49	0.68	1.35	0.80	2.29	Published	9
Jenny 2019	OS	0.68	0.67	0.40	0.67	0.35	0.11	1.12	Estimated	8
	RFS	0.54	0.22	0.36	0.22	0.41	0.21	0.77		
	DSS	0.91	1	0.87	1	NA	NA	NA		
Ivan 2021	OS	0.45	0.24	0.32	0.24	0.3	0.1	0.93	Published	8
Neal 2023	OS	0.32	0.31	0.25	0.07	0.91	0.59	1.40	Published	8

*HR* Hazard ratio, *LCI* Low confidence interval, *UCI* Up confidence interval, *NOS* The Newcastle–Ottawa Scale, *NA* Not applicable, ^a^Survival data estimated from Kaplan—Meier curves using published methodology

As shown in Table 2, all the included studies are of high quality.

### Synthesis results

#### Overall survival

**Fig. 2:**
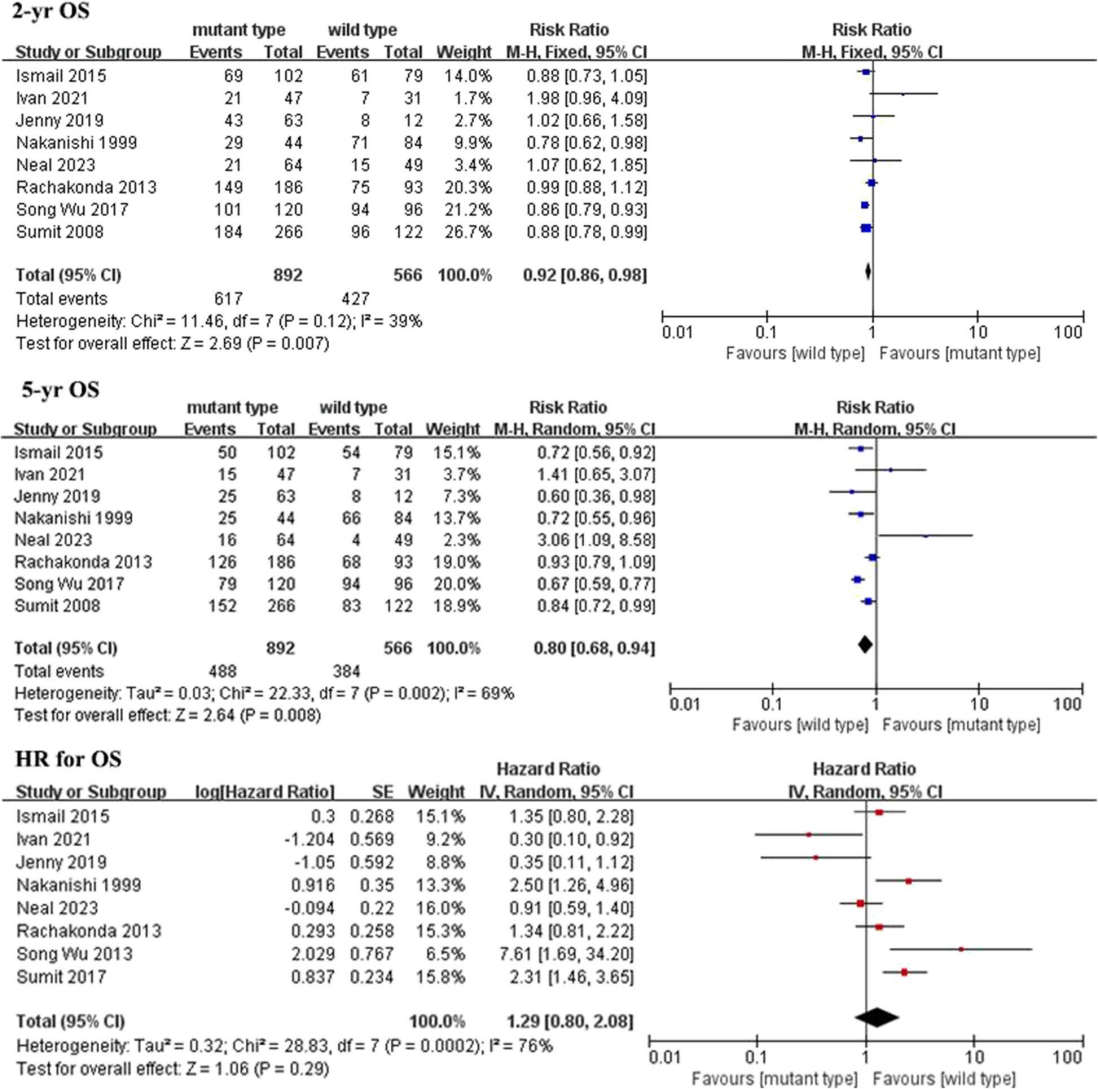
Forest plot comparing the overall survival of the *TERT* mutation and control groups

The meta-analysis included eight studies with a total of 1,458 patients [1, 6, 12–17]. Figure 2 depicts the pooled results of overall survival. Forest plots revealed that the *TERT* promoter mutation was associated with a lower 2-year OS (RR = 0.92, 95% CI 0.86–0.98; *P* = 0.007) and a lower 5-year OS (RR = 0.80, 95% CI 0.68–0.94; *P* = 0.008) compared to the *TERT* wild type. However, pooled results from eight studies showed no significant differences in the HR for OS (*P* = 0.29). These results suggest that patients without the *TERT* mutation may have a significant overall survival advantage.

#### Recurrence-free survival

**Fig. 3:**
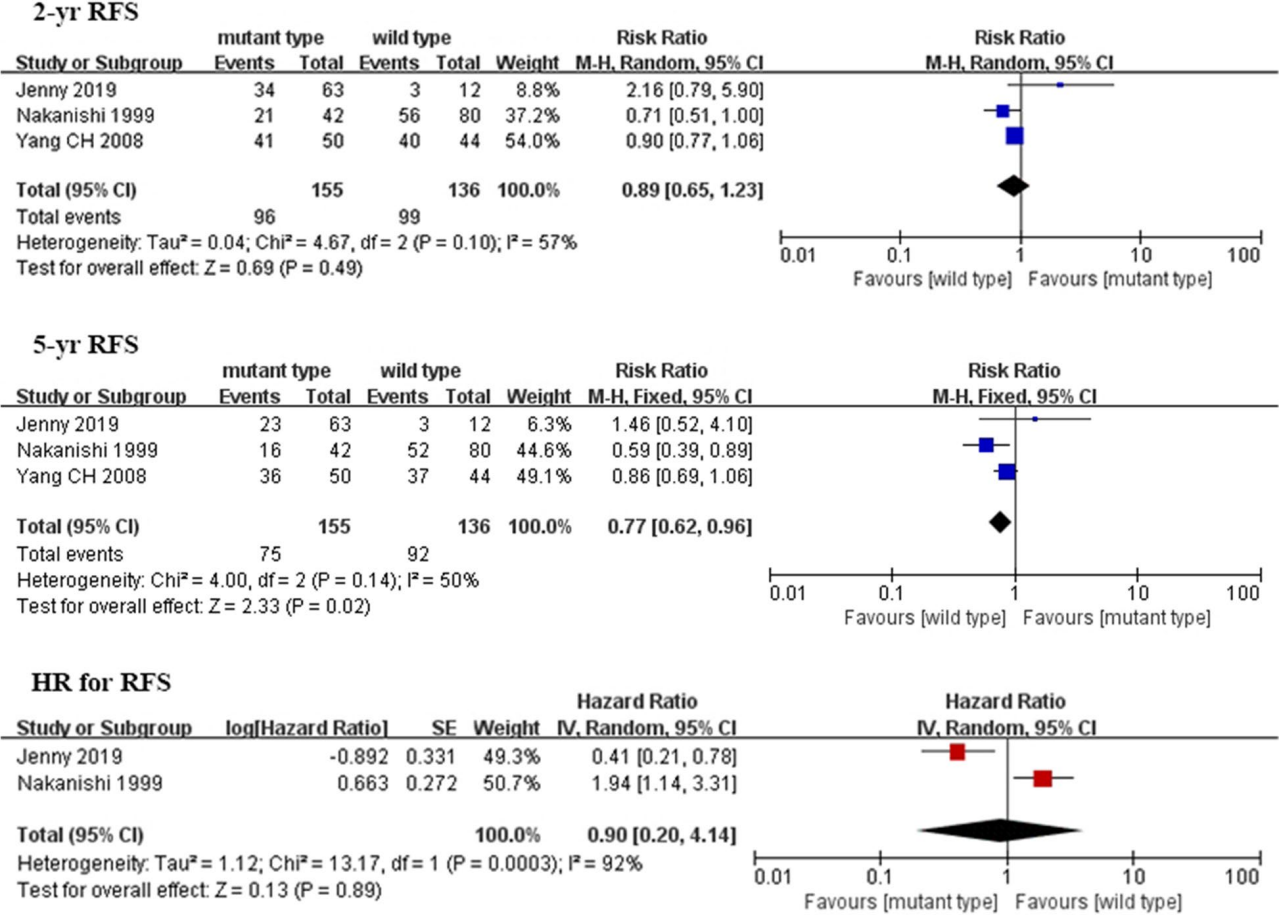
Forest plot comparing the recurrence-free survival of the *TERT* mutation and control groups

The meta-analysis included three studies with a total of 291 patients [3, 6, 14]. Figure 3 depicts the pooled results of recurrence-free survival. Meta-analysis demonstrates that the pooled estimates of 5-year RFS (RR 0.77, 95% CI 0.62–0.96; *P* = 0.02) was lower in TERT mutation group than those in control group. However, pooled results from three studies showed no significant differences in the HR for RFS and 2-year RFS. Furthermore, in the study conducted by Yang CH et al., HR for RFS was not available and thus was not incorporated into the meta-analysis. Therefore, pooled HR was not consistent with pooled 2-year and 5-year RFS.

#### Disease-specific survival

**Fig. 4:**
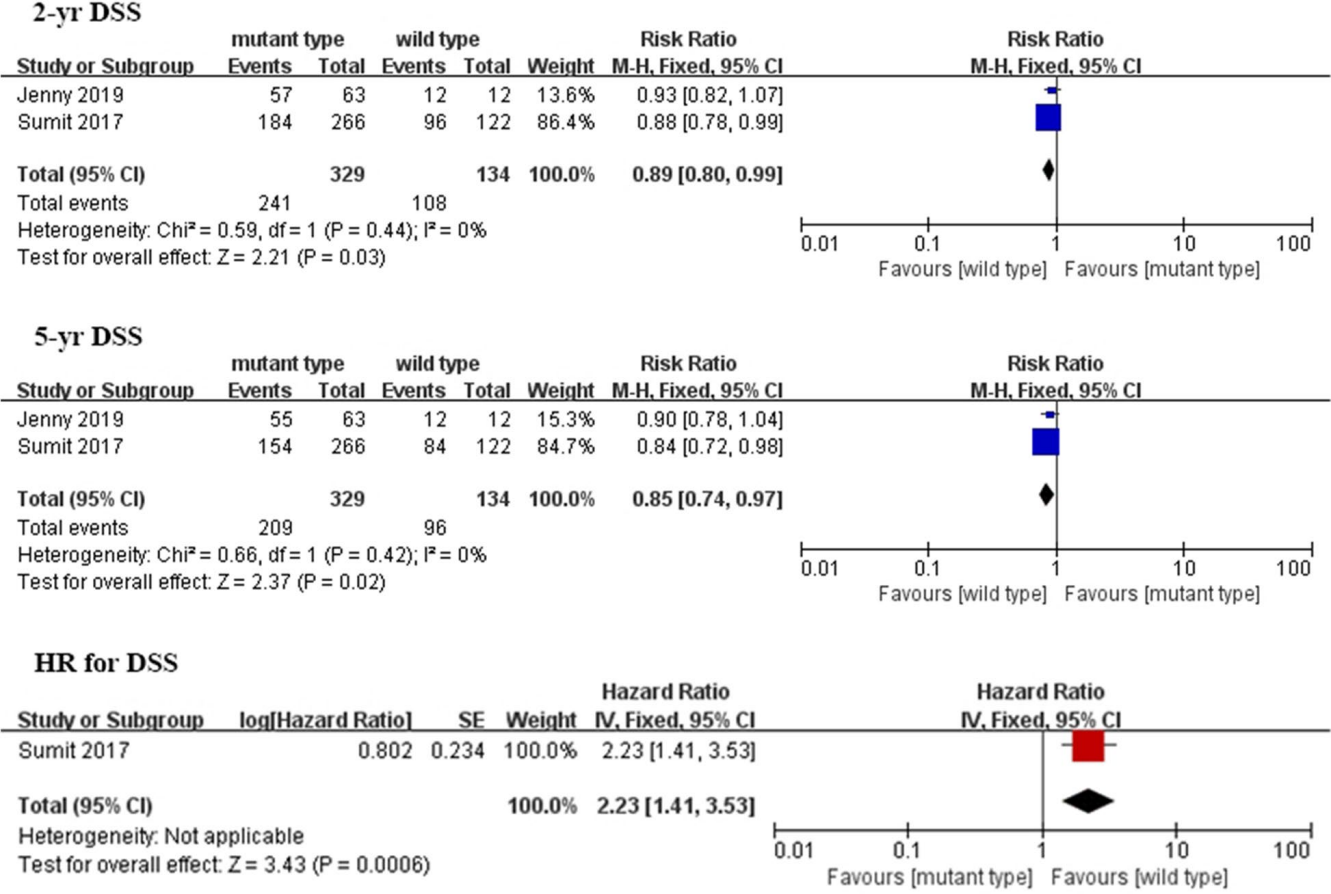
Forest plot comparing the disease-specific survival of the *TERT* mutation and control groups

Figure 4 depicts the overall pooled results of the preliminary analysis of disease-specific survival. 2-year DSS and 5-year DSS results were available from three studies with a pooled OR of 0.89 (95% CI 0.80–0.99; *P* = 0.03), 0.85 (95% CI 0.74–0.97; *P* = 0.02), respectively. And pooled HR for DSS was 2.23 (95% CI 1.41–3.53; *P* < 0.001). There was no obvious heterogeneity between the included studies, and pooled analysis showed significant differences in the 2-year DSS, 5-year DSS, and HR for DSS. Therefore, these results indicate that patients without the *TERT* mutation have a DSS advantage.

#### Sensitivity analysis and heterogeneity

Sensitivity analysis was carried out only when more than three studies were compared. When the studies conducted by Neal and Rachakonda were excluded from the OS analysis, the pooled 5-year OS was 0.74 (95% CI 0.65–0.84; *p* < 0.001). Meanwhile, the heterogeneity was also changed (*I*^2^ = 40%, *P* = 0.14). Rachakonda’s study was excluded because its outcome differed significantly from other studies (RR = 0.93, 95% CI 0.79–1.09; *p* > 0.05) and it had a high weight. Neal’s study was excluded because of analogous reasons. Furthermore, after running the leave-one-out test, the leave-one-out sensitivity analysis showed no significant differences in terms of HR for OS. Although there was still heterogeneity among the included studies, this is not surprising given global economic, cultural, and ethnic differences.

#### Assessment of publication bias

We were unable to assess publication bias because the testing ability was insufficient when there were 10 or fewer studies [23, 24].

## Discussion

We present the first systematic review and meta-analysis investigating the effect of the *TERT* mutation on the prognosis of UC patients. The analysis included nine studies with a total of 1,552 patients. OS and RFS are the most extensively studied survival outcomes that have been investigated in multiple studies. In contrast, MFS has only been the subject of one study [15].

Several key findings are reported in our pooled study analysis. First, the *TERT* mutation is found in the majority of UC patients, and some studies have demonstrated that such a mutation is an early event in the progression of UC [13]. Second, the *TERT* mutation is associated with a higher risk of mortality and recurrence, even though there is no statistical significance in the HR for OS and RFS. Third, compared to the 2-year survival rate, a more pronounced disparity is observed in the 5-year survival rates between the mutant type and the wild type. This observation signifies that UC patients with the *TERT* mutation experience an inferior long-term survival outcome. The consistency of these findings indicates their reliability and robustness as a whole.

At present, next-generation sequencing technology enables us to gain a comprehensive understanding of cancer biology and the pathogenesis of UC [25]. UC has a higher frequency of mutations than other human tumors. These include mutations of tumor suppressor genes (TP53, RB1), the RAS/RAF pathway, the mTOR pathway, and the *TERT* gene promoter [25, 26]. *TERT* mutations are typically found in two hotspots of the promoter region: chromosome 5: -124G > A and -146G > A [1, 6, 12, 13, 15]. Recent studies have linked *TERT* promoter mutations with tumorigenesis in UC. *TERT* promoter mutations also have a high potential for UC diagnosis and prognosis [27].

The *TERT* promoter mutation is one of the most common gene mutations in UC [3, 28]. In the study conducted by Boaz Kurtis et al. [28–30], *TERT* mutation status did not correlate with age, sex, tumor location, histological grade, pathological stage, or invasiveness. Therefore, *TERT* can be used as an independent predictor of prognosis in UC patients. In other words, regardless of tumor grade or stage, *TERT* can be used as a predictor of the prognosis of the tumor. However, in the study conducted by Ping Yuan et al. [31], *TERT* non-mutation carriers in cancer patients were younger than carriers, and female patients were less likely to carry the *TERT* mutation. Therefore, there is a contradiction between the studies of Boaz Kurtis and Ping Yuan. Furthermore, one study found that *TERT* promoter mutations are rarer in patients under 39 years of age [32]. This is consistent with the study conducted by Ping Yuan. However, the predictive value of *TERT* would not be affected because the age at diagnosis in UC patients is mostly greater than 60 years [2].

This review has several limitations. The relationship between the *TERT* mutation and MFS cannot be estimated due to the lack of positive events. Furthermore, the small sample size of the included studies makes it difficult to draw a reliable conclusion. In the study conducted by Sumit et al. [15], the *TERT* mutation decreased MFS in UC patients. Therefore, more high-quality research is required to evaluate the relationship between the *TERT* mutation and MFS in UC patients. Moreover, Ismail et al. showed that gender, age at diagnosis, tumor grade and stage, type of disease, and lymph node metastasis were all independently associated with poor patient survival [1, 13, 15]. Unfortunately, because some clinical information was missing in the included studies, we were unable to perform a subgroup analysis based on sex, age, tumor grade, or tumor stage. Similarly, HR values with 95% CI were missing in some studies. We used the method mentioned in the study conducted by Jayne F. Tierney [19] to calculate HR with a 95% CI. Some errors were inevitable in this process.

Additional research is required to evaluate the *TERT* gene mutation and determine its effect on the prognosis of a more explicitly defined population. This population should include patients with bladder cancer, ureteral cancer, and renal pelvis cancer. Such studies should be prospective and multicenter, and they should compare the *TERT* mutant to the wild type. These studies should include both baseline characteristics (gender, age at diagnosis, tumor stage, grade, and type of disease) and survival outcomes (OS, RFS, DSS, and MFS). In addition, although *TERT* showed clinically relevant values in urothelial carcinoma, a rapid and cost-effective method needs to be developed before routine use.

## Conclusion

In conclusion, this meta-analysis provides pooled estimates of the effect of the *TERT* mutation on the prognosis of UC patients. *TERT* mutations in UC patients are associated with poor survival and prognosis. *TERT* may be a unique marker with individual prognostic value. To further determine the effect of the *TERT* mutation on UC patients, prospective multicenter large-cohort studies are needed.

## Data Availability

All data generated or analyzed during this study are included in this published article.

## Abbreviations

TERT: Telomerase reverse transcriptase
UC: Urothelial carcinoma
OS: Overall survival
RFS: Recurrence-free survival
HR: Hazard ratio
RR: Relative risk
CI: Confidence interval
DSS: Disease-specific survival
MFS: Metastasis-free survival
CA-199: Carbohydrate antigen 19–9
UTUC: Upper tract urothelial cancer
